# HiFocus Helix™ electrode insertion: surgical approach

**DOI:** 10.1186/s13104-015-1267-9

**Published:** 2015-07-15

**Authors:** Arthur Menino Castilho, Henrique Furlan Pauna, Fernando Laffitte Fernandes, Rodrigo Gonzales Bonhin, Alexandre Caixeta Guimarães, Tatiana Mendes de Melo, Margareth Cheng, Edi Lucia Sartorato, Guilherme Machado de Carvalho, Jorge Rizzato Paschoal

**Affiliations:** Otology, Audiology and Implantable Ear Prostheses, Ear, Nose, Throat and Head and Neck Surgery Department, State University of Campinas, UNICAMP, PO BOX 6111, São Paulo, 13081-970 Brazil

**Keywords:** Cochlear implant, Perimodiolar electrode, HiFocus Helix™ electrode

## Abstract

**Background:**

Cochlear implants have been used for almost 30 years as a device for the rehabilitation of individuals with severe-to-profound hearing loss. One of the important aspects of cochlear implantation is the type of electrode selected and proper insertion of the electrode array in scala tympani to minimize cochlear damage. The HiFocus Helix™ electrode is a precurved design aimed at placing the electrode contacts close to the spiral ganglion cells in the modiolus. The prescribed insertion techniques are intended to minimize the likelihood of damage to the basilar membrane or lateral wall of the cochlea.

**Case presentation:**

To describe the first insertion of a HiFocus Helix™ electrode in Brazil exposing surgical particularities and device details in a patient with profound hearing loss, due to Mondini’s dysplasia.

**Conclusion:**

No problems were encountered during the surgical procedure. The patient experienced improvement in hearing thresholds and speech perception. The HiFocus Helix™ electrode proved easy to insert and provided expected hearing benefits for the patient. This manuscript indicates that the HiResolution™ Bionic Ear System with HiFocus Helix™ electrode comprise a cochlear implant system that is practical and beneficial for the treatment of severe-to-profound hearing loss.

## Background

Cochlear implants are surgically implanted devices that provide hearing sensation to individuals with severe-to-profound hearing loss who obtain limited benefit from hearing aids. By electrically stimulating the auditory nerve directly, a cochlear implant bypasses damaged or undeveloped sensory structures in the cochlea, thereby providing usable information about sound to the central auditory nervous system. Over the last 30 years, cochlear implants have become standard treatment for adults and children with severe-to-profound profound bilateral sensorineural hearing loss.

In particular, hearing impairment in very young children has a significant impact on both language and social development. The prevalence of congenital hearing loss varies from 1.2 to 2.7 per 1,000 newborns. Recent information estimates a current rate of 1.4 per 1,000 newborns in the United States, which makes it the most frequent sensory deficit present at birth [1]. Fortunately for these young children, cochlear implants can provide the opportunity to develop hearing and speech abilities that is close to peers with normal hearing [2].

One of the important components of a cochlear-implant system is the electrode array that is inserted surgically into the cochlea. Electrode technology has evolved since the first cochlear implants were introduced in the early 1980s. Contemporary electrode designs are aimed at (1) limiting intracochlear damage during electrode insertion, and (2) reducing the current required for usable hearing [3].

The HiFocus Helix™ electrode [4] (Advanced Bionics LLC, USA) addresses the first two of those aims. Its perimodiolar design places the stimulating contacts near the spiral ganglion cells in the cochlear modiolus. Proximity to the ganglion spiral cells is expected to provide better stimulation at lower current levels, thus resulting in lower power requirements.

Several studies have shown that the HiFocus Helix™ meets its design goals. A temporal bone study was conducted to analyze a prototype of the Helix II™ electrode [5]. The Helix II™ was shown to have a 436-degree insertion angle and cochlear medial wall positioning. No evidence of inner ear trauma in the temporal bones was identified. A prospective CT-scan analysis of 39 HiFocus Helix™ electrode recipients showed correct placement of the electrode into scala tympani in 100% of cases when utilizing an electrode insertion tool and in 85.7% of cases without the insertion tool [6].

The aim of this study is to describe the first insertion of a HiFocus Helix™ electrode in Brazil. Surgical particularities, device details, and its benefits are discussed.

## Case presentation

This article is based on the description of the surgical technique of pre-curved electrode insertion (Advanced Bionics HiFocus Helix™). Medical records of the patient selected as well as a review of the literature were analysed.

### Device

**Figure 1:**
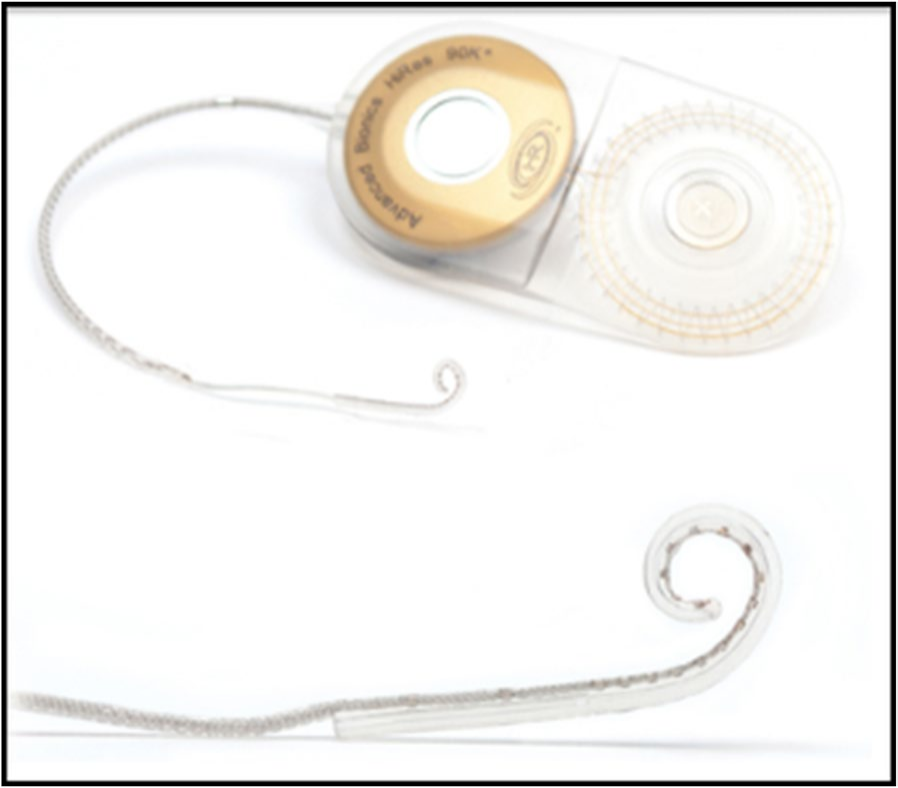
HiFocus Helix electrodes (from HiFocus Helix electrode surgical guide). Permission to republish images granted by copyright holders Advanced Bionics.

**Figure 2:**
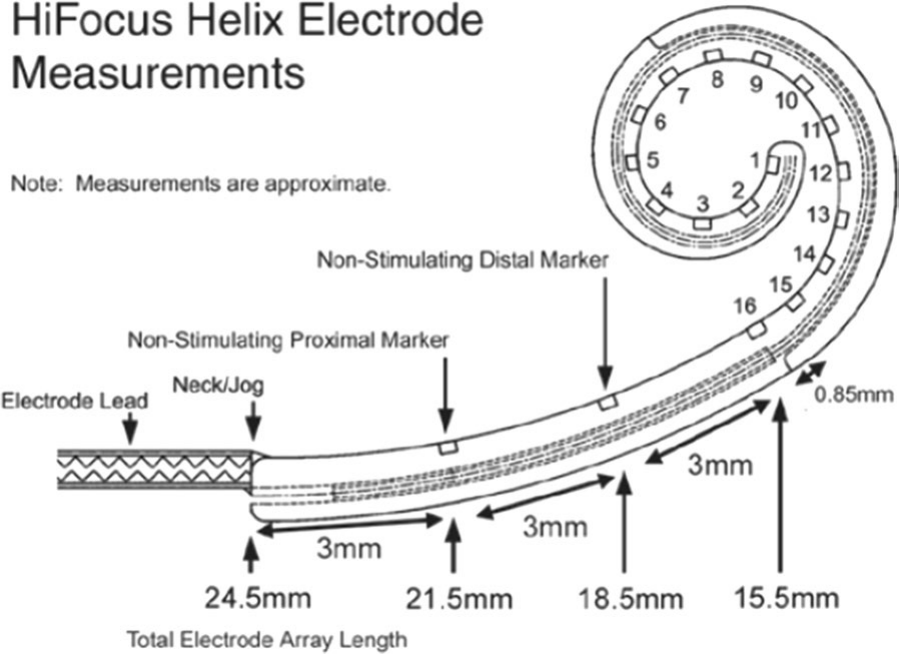
Measurements of HiFocus Helix electrode (from HiFocus Helix electrode surgical guide). Permission to republish images granted by copyright holders Advanced Bionics.

The HiResolution™ Bionic Ear cochlear implant system (Advanced Bionics LLC, USA) was selected for this patient. It was used the HiRes 90K™ implant with HiFocus Helix™ electrode implantable components (Figure 1). The HiFocus Helix™ electrode is a highly pre-curved device that is designed for close perimodiolar placement. It has a total length of 24.5 mm with 16 stimulating contacts that face the modiolar wall for highly focused and selective stimulation of the spiral ganglion cells (Figure 2). The HiFocus Helix™ is intended to be inserted 360°–430° into the cochlea using a cochleostomy of 1.2–1.6 mm (Advanced Bionics).

### Subject

The patient was a 17-year-old female referred to our hospital for the evaluation of candidacy for a cochlear implant. Her hearing loss was diagnosed at the age of 5 years and the patient began using bilateral hearing aid 1 year later. There were no abnormalities observed at a physical examination.

**Figure 3:**
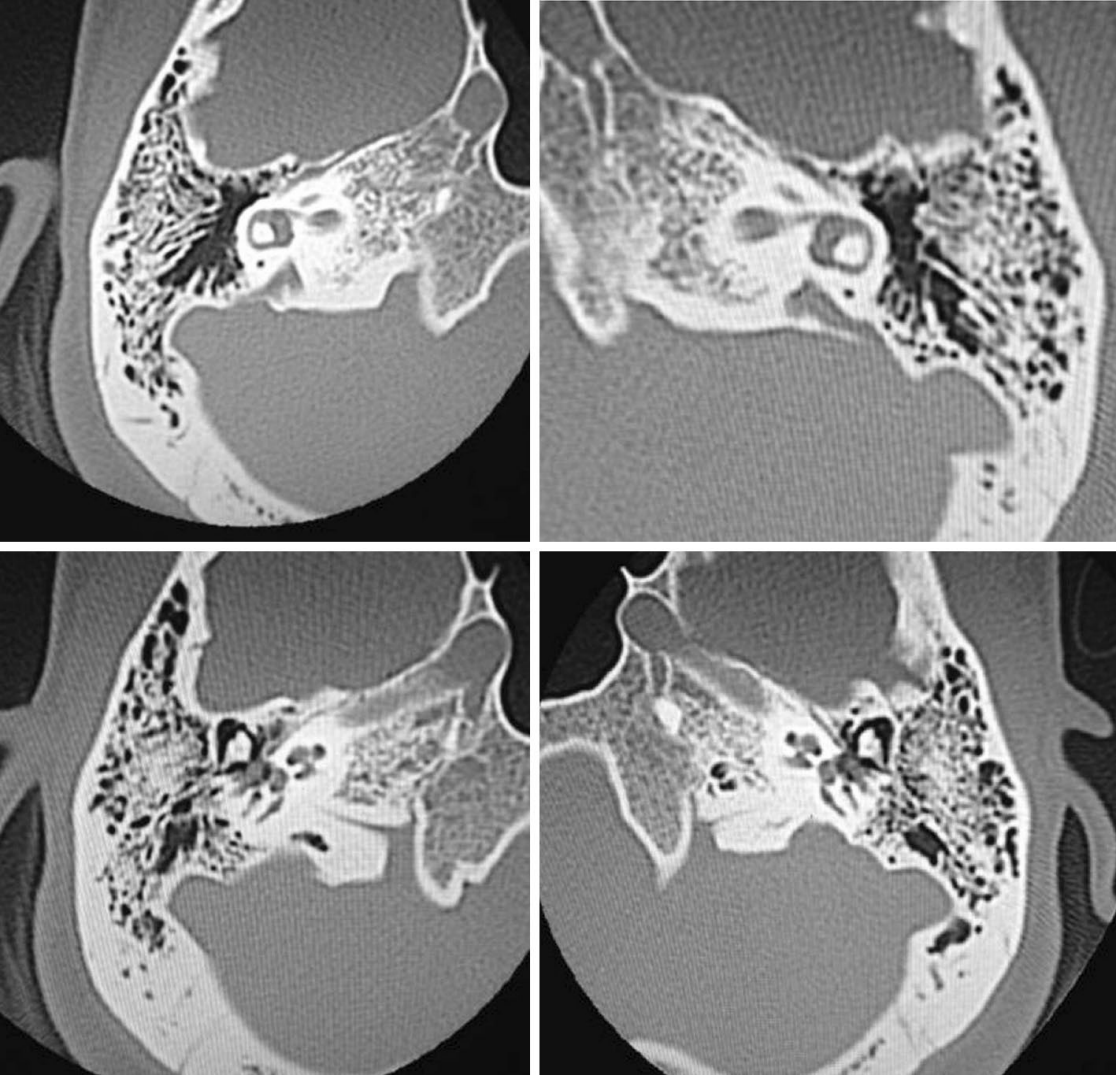
CT scan, axial section, there is an enlarged vestibular aqueduct and malformation of the cochlea, corresponding to Mondini dysplasia in both sides.

High resolution temporal bone CT and MRI studies detected cochlear malformation and large vestibular aqueduct in both sides, thus characterizing Mondini dysplasia (Figure 3).

Before surgery, the patient was informed about the surgical procedure, implant device, post-operative expectations, and possible complications.

### Surgical technique

The HiRes 90K™ implant and HiFocus Helix™ electrode were surgically implanted using manufacturer-recommended techniques and procedures (Advanced Bionics). Facial nerve function was monitored during surgery using the Xomed-Treace Nerve Integrity Monitor-2 (NIM 2™). The monitoring electrodes were placed in the frontal and zygomatic areas. No problems were encountered during surgery and no complications occurred.

**Figure 4:**
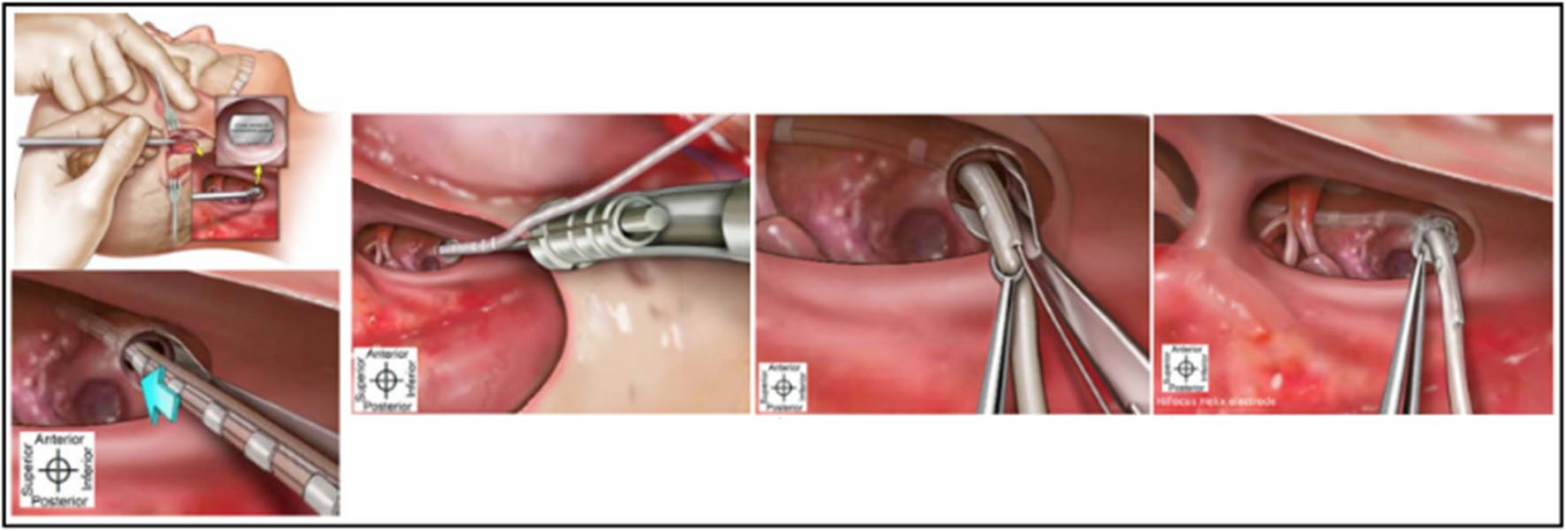
Insertion method of the HiFocus Helix electrode (from HiFocus Helix electrode surgical guide). Permission to republish images granted by copyright holders Advanced Bionics.

**Figure 5:**
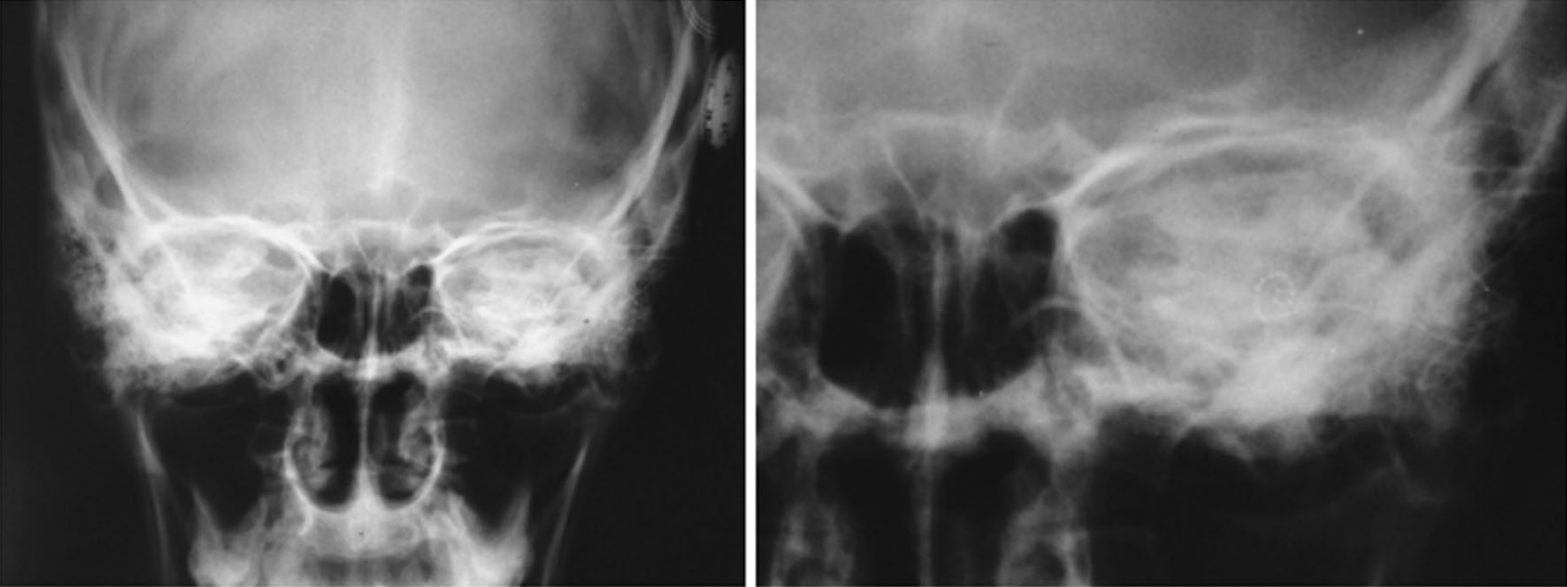
Post-operative transorbital X-Ray. Observe the positioned electrode inside the left cochlea.

The patient was under general anesthesia and placed in supine position. Antibiotic prophylaxis (Cefazolin 1 g) was administered intravenously. The surgical steps are described below:

1. Antisepsis of the entire face and retroauricular region;
2. Retroauricular trichotomy, micropore tape used to isolate this region;
3. Local anesthesia (Lidocaine 2% with epinephrine, applied dosage of 4% mg lidocaine/kg) in retroauricular area;
4. New antisepsis of the mentioned regions;
5. Placement of sterile sheets in retroauricular areas;
6. Rectilinear retroauricular incision of approximately 4 cm and dissection along anatomical places; preparation of a cross-like muscle-periosteal flap;
7. Removal of small fragments of fascia and temporal muscle to occlude the cochleostomy;
8. Simple mastoidectomy, identifying the lateral semicircular canal, the short process of the incus, the posterior wall of the outer ear canal, tegmen tympani and the lateral sinus, gathering a small amount of bone dust;
9. Thinning of the posterior wall of the outer ear canal, posterior tympanotomy with preservation of the incus buttress;
10. After identification of the round window niche, a 1.5 mm cochleostomy is created antero-inferior to the round window;
11. Placement of internal part of the device in a periosteal pocket created in the squamous part of the temporal bone;
12. Insertion of the electrode into the cochleostomy using and electrode inserter;
13. Positioning the muscle graft around the electrode to seal the cochleostomy; placing bone dust to close the posterior tympanotomy;
14. Closure with Vycril 3.0 sutures on the muscle-periosteum flap and subcutaneous tissue; skin closure with Nylon 4.0;
15. Cleaning of the patient and external compressive dressing;
16. Impedance testing, neural response imaging (NRI) and a transorbital incidence radiograph to confirm intracochlear position of the electrode (Figures 4, 5).

## Results

We experienced no problem during the electrode insertion and we observed an improvement in hearing thresholds and speech perception of the patient.

## Conclusion

As cochlear implantation benefits have been well established, a new focus has been directed to better performance devices combined with less trauma to the inner ear. In this concept, the Helix model was created using a perimodiolar positioning that would provide better neural stimulation [4–8].

First perimodiolar electrodes were introduced 12 years ago, and can be either curved or designed to curve at itself during insertion. Proximity to the ganglion spiral cells is sought to provide better stimulation at lower current levels, thus leading to less cochlear damage and less energy consumption [4–8].

A temporal bone anatomic study was conducted to analyze prototypes of models Thin Lateral™ and Helix II™ (Advanced Bionics Corporation). The study observed a medium depth of insertion of 368 and cochlear lateral wall positioning in the Thin Lateral™ model. Helix II™ was shown to have a 436 insertion and cochlear medial wall positioning. No evidence of inner ear trauma in the temporal bones was identified for both devices. The lesser diameter in these devices compared to conventional electrodes seems to contribute to performance and less cochlear trauma [5, 6].

The curved shape and lateral flexibility of the Thin Lateral™ device allows the bundle of electrodes to follow the cochlear lateral wall curvature, therefore avoiding damage and risk of tympanic scale manipulation [6, 8].

The Helix II™ model has an insertor that is removed during insertion allowing the bundle of electrodes to curve medially along the tympanic scale, diminishing the risk of trauma to the spiral ligament [6].

Another prospective analysis with 82 patients aged between 18 and 84 years fitted with devices Nucleus^®^ Freedom™ RECA (Cochlear^®^) and Advanced Bionics HiRes 90K™ HiFocus Helix™ (Advanced Bionics) showed a correct positioning of the electrodes through computed tomography in 95.1% of the cases. All cases were implanted via round window with or without an insertor. The radiologic confirmation of the electrode position inside the tympanic scale was obtained in 95.4% of cases fitted with Nucleus^®^ Freedom™ device. The Helix 90k™ device showed correct placement in 100% of cases when utilizing an electrode insertor and 85.7% without the insertor [5].

The perimodiolar HiFocus Helix™ electrode proved easy to insert in this patient. In addition, the recipient was able to experience better hearing thresholds and improved speech perception compared to pre-operatively with a hearing aid. Thus, the HiFocus Helix™ electrode is a viable and effective electrode option for patients who choose a HiResolution™ Bionic Ear.

## Consent

Written informed consent was obtained from the patient for publication of this Case Report and any accompanying images. A copy of the written consent is available for review by the Editor-in-Chief of this journal. Ethical precepts of the institution were respected (CEP 011/2013, Fátima Aparecida Böttcher Luiz, MD, Ethics Committee coordinator).
